# A unified scheme for the benchmarking of upper limb functions in neurological disorders

**DOI:** 10.1186/s12984-022-01082-8

**Published:** 2022-09-27

**Authors:** Valeria Longatelli, Diego Torricelli, Jesús Tornero, Alessandra Pedrocchi, Franco Molteni, José L. Pons, Marta Gandolla

**Affiliations:** 1 Neuroengineering and Medical Robotics Laboratory and WE-COBOT Laboratory, Department of Electronics, Information, and Bioengineering, Politecnico di Milano, Milan, Italy; 2 Neural Rehabilitation Group, Cajal Institute, Spanish National Research Council (CSIC), Madrid, Spain; 3 Advanced Neurorehabilitation Unit, Hospital Los Madroños, Madrid, Spain; 4 Villa Beretta Rehabilitation Center, Valduce Hospital, Costa Masnaga, Italy; 5 Shirley Ryan AbilityLab, Chicago, USA; 6 WE-COBOT Laboratory, Department of Mechanical Engineering, Politecnico di Milano, Milan, Italy

**Keywords:** Exoskeletons, Benchmark, Testing, Performance evaluation, Rehabilitation robotics, Functional evaluation, Upper limb, Neurological disorders, Stroke

## Abstract

**Background:**

In neurorehabilitation, we are witnessing a growing awareness of the importance of standardized quantitative assessment of limb functions. Detailed assessments of the sensorimotor deficits following neurological disorders are crucial. So far, this assessment has relied mainly on clinical scales, which showed several drawbacks. Different technologies could provide more objective and repeatable measurements. However, the current literature lacks practical guidelines for this purpose. Nowadays, the integration of available metrics, protocols, and algorithms into one harmonized benchmarking ecosystem for clinical and research practice is necessary.

**Methods:**

This work presents a benchmarking framework for upper limb capacity. The scheme resulted from a multidisciplinary and iterative discussion among several partners with previous experience in benchmarking methodology, robotics, and clinical neurorehabilitation. We merged previous knowledge in benchmarking methodologies for human locomotion and direct clinical and engineering experience in upper limb rehabilitation. The scheme was designed to enable an instrumented evaluation of arm capacity and to assess the effectiveness of rehabilitative interventions with high reproducibility and resolution. It includes four elements: (1) a taxonomy for motor skills and abilities, (2) a list of performance indicators, (3) a list of required sensor modalities, and (4) a set of reproducible experimental protocols.

**Results:**

We proposed six *motor primitives* as building blocks of most upper-limb daily-life activities and combined them into a set of functional *motor skills*. We identified the main aspects to be considered during clinical evaluation, and grouped them into ten *motor abilities* categories*.* For each ability, we proposed a set of *performance indicators* to quantify the proposed ability on a quantitative and high-resolution scale. Finally, we defined the procedures to be followed to perform the benchmarking assessment in a reproducible and reliable way, including the definition of the kinematic models and the target muscles.

**Conclusions:**

This work represents the first unified scheme for the benchmarking of upper limb capacity. To reach a consensus, this scheme should be validated with real experiments across clinical conditions and motor skills. This validation phase is expected to create a shared database of human performance, necessary to have realistic comparisons of treatments and drive the development of new personalized technologies.

## Introduction

Neurological damages following stroke, spinal cord injury, and other neurological or neurodegenerative disorders can result in severe impairment of sensorimotor functions, affecting functional activities, independence, and eventually the quality of life. This is particularly true for the upper extremities, which are fundamental to interact with the environment and perform activities of daily living [1].

In the context of neurorehabilitation, assessing upper limb movements is crucial to monitor and understand sensorimotor recovery [2]. Technology-aided assessments could provide the clinicians with objective, accurate, and repeatable measurements of a patient’s capacity, allowing them to monitor his/her progress objectively, evaluate the effects of the different treatments or adapt them to the specific patient’s needs [3]. Nevertheless, so far, the evaluation of limb functions and the assessment of the effectiveness of technology-assisted interventions have relied mainly on clinical scales [4, 5]. Clinical scores applied to the upper limbs have several drawbacks, such as relying on observer-based ordinal scales (e.g., Functional Independence Measure), having poor inter-rater and intra-rater reliability, and floor and ceiling effects (e.g., Fugl-Meyer Assessment) [6–8]. Consequently, they also often fail to differentiate between improvements at motor recovery level and improvements due to alternative compensating strategies [9].

Many instrumented approaches, including kinematics, electromyography (EMG), or brain activity analysis, can be exploited to support the subjective evaluation performed by the clinician, enhance the understanding of the patient’s improvement, and provide a better understanding of the relationship between the mechanisms of cortical reorganization and motor recovery [10–12]. These measurements are commonly named biomarkers. Sensor-based approaches, considering, for example, optoelectronic systems, inertial measurement units, or EMG sensors, have been shown to apply to various tasks [1, 7]. Recently, robotic devices, such as exoskeletons, have emerged as a novel solution for assessing movement behavior during an intervention, exploiting data acquired by the integrated sensors [13, 14]. Robots allow recording and analyzing measures concurrently from multiple joints during a well-controlled and highly repeatable task. Moreover, they can actively perturb the patient’s movement to investigate neuromuscular control and related dysfunctions [2].

In the last years, hundreds of studies have exploited biomarkers to evaluate limb capabilities, assess the efficacy of rehabilitation interventions, or understand the implications of using robotic devices for rehabilitation. This resulted in a plethora of potentially helpful evaluation methods and protocols [11, 15–18]. This variety of quantitative outcome metrics is particularly noticeable for the upper limb functions, being the target functions more varied and complex than for the lower limb, where both gait protocols and sensors-based outcome measures are more established and recognized in clinical and research contexts.

In recent years, we are observing a growing awareness of the importance of benchmarking [19]. Benchmarking can be defined as *standardized evaluation.* It consists in measuring the performance of a system with a set of metrics, which are then compared to a set of standards or points of reference, namely the benchmarks. The adoption of benchmarking promotes the development and use of standardized and reproducible tests able to provide quantitative evaluation and comparison of systems [20]. So far, its application to the neurorehabilitation field is still missing [15].

Systematic benchmarking methodologies have been recently promoted by two European initiatives: the EUROBENCH project “European Robotic Framework for Bipedal Locomotion Benchmarking” ([21], http://www.eurobench2020.eu/), and the EU COST Action CA16116 “Wearable Robots for Augmentation, Assistance or Substitution of Human Motor Functions” (https://www.cost.eu/actions/CA16116). The EUROBENCH project developed the first benchmarking scheme for lower-limb exoskeletons and prostheses, creating a sustainable “benchmarking infrastructure” composed of a testing facility and a set of algorithms and metrics able to quantify a wide spectrum of motor abilities related to bipedal functions [19]. The EU COST Action triggered a European-wide discussion on the evaluation of the upper extremities in neurorehabilitation using technology [22]. Nevertheless, EU COST Action only provided general guidelines for the best practice regarding upper extremities evaluation without proposing a real benchmarking procedure.

While for lower limb functions, some ongoing researches have already adopted or proposed benchmarking methods [23–26], in the upper limb field, the benchmarking approach is still missing [3, 22, 27, 28].

This work aims to develop the first benchmarking framework for evaluating upper limb capabilities in clinical and research settings. The proposed scheme includes: (1) a taxonomy that identifies and classifies the relevant upper limb motor skills and motor abilities, (2) a selection of outcome measures and performance indicators able to quantify each motor ability, (3) the required sensor networks to extract the outcome measures, and (4) a set of standardized protocols that should be followed to obtain comparable results. The potential application of this benchmarking scheme is twofold: (1) to perform an instrumented evaluation of the upper limb capabilities of a subject with a neurological or neurodegenerative disorder, and (2) to assess the effectiveness of rehabilitative interventions by analyzing patients’ motor performance at different checkpoints (e.g., before and after treatment).

## Methods

This benchmarking scheme aims to evaluate neurologic and neurodegenerative disorders that cause upper limb impairments. It is focused on the upper extremity body parts, including shoulder, elbow, and wrist. It has been designed to be feasible, reproducible, transferrable, and clinically meaningful in order to be shared among the scientific and clinical communities.

**Fig. 1:**
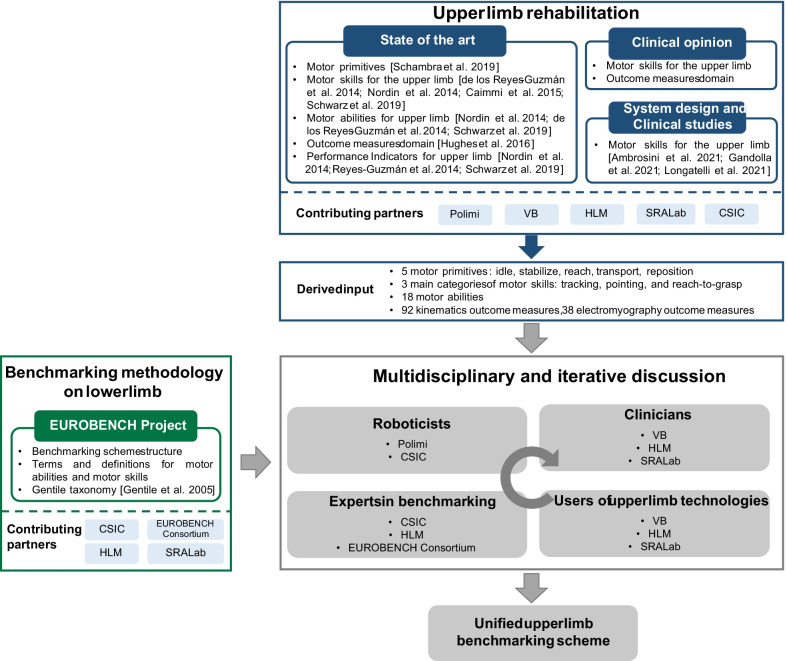
Benchmarking scheme process definition and partners’ contribution. Polimi = Politecnico di Milano; VB = Villa Beretta Neurorehabilitation Center; HLM = Hospital Los Madroños; SRALab = Shirley Ryan AbilityLab; CSIC = Consejo Superior de Investigaciones Científicas

The decision-making process to create this scheme was based on a multidisciplinary and iterative discussion among six partners with direct experience in different areas. In particular, the starting point was an extensive literature analysis on benchmarking methodologies and upper limb evaluation in clinical settings. Starting from the literature, we put together previous effort and expertise in benchmarking methodologies for human locomotion with medical knowledge and clinical experience in upper limb rehabilitation. In particular, this process involved 11 people from different institutions and more than 60 European entities participating as Beta Tester in the EUROBENCH Project, including roboticists, clinicians, experts in benchmarking, users of upper limb technologies, and engineers. The benchmarking scheme process definition and the contribution of each partner are represented in Fig. 1.

### Motor skills

The starting point of this benchmarking scheme is taxonomy. We adapted three existing taxonomies, i.e., the one proposed by Schambra and colleagues [29] for the definition of *motor primitives*, the one introduced by Magill and Anderson [30] for the definition of *motor skill* and *motor abilities,* and the one suggested by Gentile [31] for the classification of *motor skills*.

A *motor skill* can be defined as a “functional and goal-oriented activity or task” [30]. Motor skills are diverse, given the variety of interacting objects and goals, e.g., “drinking from a glass” or “moving a book”. Nevertheless, they can be considered as a combination of a limited array of building block motions called *motor primitives* [29]. The segmentation of complex movements into motor primitives is widely adopted to analyze and assess movement quality in clinical settings [1, 32, 33]. It could allow more precise tracking of the neural organization after brain injuries, since motor control and learning are believed to be neurally mediated at the level of primitives [32]. Moreover, if the patient is unable to complete the entire functional movement, the assessment of primitives can provide a more nuanced picture of the condition [29, 29]. The literature has highlighted that the main tasks performed in clinical settings for rehabilitative or evaluation purposes can be classified as tracking, pointing, and reach-to-grasp tasks [7, 9, 16, 35]. Starting from this basis, we combined motor primitives to create motor skills that fulfill the following requirements. Motor skills have to be functional [36], they must be suitable for patients from slight to severe impairments, and they should target movements usually performed in clinical settings and daily life activities [37].

In order to propose a scheme feasible also with robots, the motor skills proposed in this scheme are restricted to the sitting position and involve only one arm. We organized the motor primitives according to the Gentile’s taxonomy [31], classifying them according to two factors: (1) the *environment*, which includes the external disturbing elements interacting with the person during the execution of the motor primitive, and (2) the *function*, which specifies the functional goal of the movement [29].

### Motor abilities

The term ability has been used differently in the literature. We relied on the taxonomy of Magill and Anderson [30], which defines ability as “the capacity of an individual that determines their achievement potential to perform a specific (motor) skill”.

Several motor abilities can describe upper limb functionalities in neurological patients [9, 16, 35]. Neither consensus nor a common taxonomy has been proposed yet in the scientific literature or in the clinical domain. Based on different previous literature reviews [9, 16, 35], we selected a group of motor abilities that could be used to describe the performance of upper limb motor skills comprehensively. We defined new ones when we could not find any good candidates in the literature, e.g., in the case of motor abilities related to muscle activity.

Finally, we identified the most relevant *outcome measures domain* that should be used to quantify the proposed motor abilities. For this choice, we based on the results of the survey of the EU COST Action CA16116 [22] and on the experience of the involved clinical centers. As a trade-off between evaluation completeness and set-up time, we selected the two outcome domains that obtained the higher consensus.

### Performance indicators

of the third step of our benchmarking scheme involves the identification of *performance indicators* (Pis), defined as “outcome measures that allow the quantitative assessment of a motor ability” [30]. For each motor ability identified, we selected from literature reviews the PIs that respected at least one of the following requirements: (1) are suitable to describe the cause of upper limb impairments, (2) are correlated with standard clinical scales, or (3) have been used to assess the effect of rehabilitative interventions or for the control of upper limb devices. We included PIs that could be computed independently of the measurement system. Each PI was correlated to a motor ability. For each motor ability, we identified as “mandatory” the PIs that, according to the literature, have either the maximum correlation with the Fugl-Meyer Assessment scale, which is the most adopted primary outcome of clinical studies in neurorehabilitation. These PIs should always be included in the evaluation. The others PIs were classified as “recommended”.

### Benchmarking protocol

Establishing unified protocols is one of the major challenges and probably the primary goal in benchmarking research [19]. This last section deals with the definition of standardized procedures to be followed to perform a reproducible and reliable benchmarking assessment.

## Results

### Motor skills

Inspired by the work of Schambra and colleagues [29], we defined six motor primitives: idle, stabilize, point-to-point reach, reach for grasp, transport, and reposition (Table 1). The definition of these motor primitives was based on the decomposition into constituent primitives of activities of daily life activities, whose validity and reliability were previously assessed on healthy subjects and post-stroke patients [29]. We deviated from Schambra’s work [29] for what concerned the motor primitive “reach”. In particular, we distinguished between point-to-point reach, i.e., reaching a target point with the hand without contact with any object, and reach for grasp, i.e., if the subject is asked to grasp an object in the conclusive part of the task. Indeed, it has been demonstrated that the reaching movement is different depending on the type of movement foreseen after the reaching phase and, according to the specific goal to be achieved, the action planning and the kinematics patterns are different [39, 40]. In this work, we considered only the palmar grasping of an object of cylindric shape, as will be detailed in “Benchmarking protocol” section. We neglected the variety of possible grasping strategies which can affect the arm motor plan, given that this aspect is beyond the goal of the present study.

**Table 1:** Upper limb motor primitives

Motor Primitive	Definition
Idle	Holding the upper limb in a stable position without contact with any object
Stabilize	Holding a target object still. There is the grasp of a target object throughout the minimal-motion
Point-to-point reach	Reaching a target point without contact with any object
Reach for grasp	Reaching a target object and make contact with it through grasping
Transport	Moving a target object in space
Reposition	Moving away from the target object toward the idle position, without contact with any other object

The identified motor primitives were combined to define the following three main motor skills, which represent the most common activities considered in clinical evaluation [9, 16, 35, 41]: *anterior reaching*, *moving objects*, and *hand to mouth.* A detailed description of motor skills is provided in Sect. 3.4.

We adapted Gentile’s taxonomy to classify the six motor primitives. Considering the environment, the main discriminant is the execution of the movement in the presence or not of gravity [42]. We classified the environment into *micro-gravity* (i.e., when tasks are executed with the arm suspended or supported by any tool, or performed on the plane considering negligible friction) and *gravity* (i.e., when tasks are performed without the aid of external support systems). Each environment category contains both upwards and downwards movements. Each one is then subdivided into two subcategories based on the absence or presence of a *disturbance*. Examples of disturbances could be a payload, a cognitive dual-task, or external forces. The disturbance might be defined according to a specific clinical/scientific question but must be quantitatively specified before applying the protocol, and it must be replicable.

As for the function, we distinguished between upper limb *stability*, if the goal is to maintain the arm location unchanged for more than 1 s, and upper limb *transport* otherwise [29]. This time interval corresponds to the mean duration of upper limb ADLs [37]. Each one was in turn subdivided into two categories: without object manipulation and with object manipulation.

**Fig. 2:**
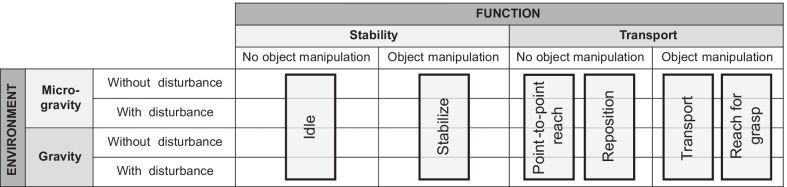
Taxonomy for classifying upper limb motor primitives involved in the upper limb benchmarking scheme

The identified motor primitives can be represented as in the schema shown in Fig. 2.

### Motor abilities

We defined a set of ten motor abilities (Table 2): *accuracy, efficacy, efficiency, movement amplitude, muscular effort, intra-limb coordination, planning predictability, power, smoothness,* and *speed*. Power and muscular effort abilities, as well as their definitions, were introduced for the first time in this work. The other abilities and relative definitions were, instead, identified from the literature [9, 16, 35]. We did not consider abilities for bilateral tasks since this scheme addresses only one limb. For the sake of conciseness, we included temporal abilities (i.e., temporal posture and temporal efficiency) in other more general categories (intra-limb coordination and efficiency, respectively), and we unified precision and accuracy into one motor ability (i.e., accuracy).

**Table 2:** Upper limb motor abilities

Motor Ability	Description	Outcome measure domain		Source
		Kinematics	EMG	
Accuracy	Spatial error of movements relative to optimal behavior	x		[9, 16, 35]
Efficacy	Successful achievement of a targeted task goal	x		[9, 16, 35]
Efficiency	Quality of how a targeted task goal is reached	x	x	[9, 16, 35] Adapted for EMG domain
Movement amplitude	Maximally reachable area or volume with a specific joint and position-related aspects of single or multiple joints	x		[9, 16, 35]
Muscular effort	Muscular activation associated with the production of muscle tension while achieving a task		x	//
Intra-limb coordination	Correlation and redundancies in upper limb joints that produce different strategies to complete the task	x	x	[9] Adapted for EMG domain
Planning predictability	Ability to perform goal-directed movements in a feedforward manner	x	x	[9, 16, 35]
Power	Ability to produce force or power while performing exercises		x	//
Smoothness	Quality of feedforward control based on the deviation of the velocity profile to an optimal, bell-shaped velocity profile	x	x	[9, 16, 35]
Speed	How fast movements are performed	x		[9, 16, 35]

*EMG* = *Electromyography; // indicates that the ability has not been previously defined in the literature*

Each ability could be associated with upper limb impairment. In particular, *accuracy* and *efficacy* could quantify the paresis, *efficiency*, *intra-limb coordination*, and *movement amplitude* could be correlated to a loss or regain of fractionated movements, *planning predictability* could be associated with a loss or regain of somatosensation, and, finally, *muscular effort*, *power*, *speed,* and *smoothness* could reflect the muscle tone [9, 38].

Finally, the *outcome measures domains* included in this scheme were *kinematics* and *electromyography*. Indeed, according to the results of the EU COST Action CA16116 [22], these were the two domains that obtained the higher consensus by both clinicians and researchers as essential to be included in the assessment procedures. Kinematic variables are used to capture the degree of motor impairments through objective, precise, and detailed measurements of movement performance and quality [43]. They can describe feedforward sensorimotor control [9], reveal compensatory strategies [35], describe selective motor control [44], and quantify upper limb workspace and coordination [9]. Therefore, they are suitable measures to describe movement dysfunctions, and they have been extensively reported [11, 17, 43, 45, 46]. Kinematics can be acquired with optoelectronic systems or inertial sensors, whose use is nowadays diffused in the clinical setting and research laboratories, or using encoders of the robot when available. Considering electromyography, the scientific community recognizes EMG-based measures as key for quantifying muscle activation in terms of motor unit recruitment capability [47], fatigue [48], synergies [49], co-contractions [50], and indirect investigation of neural plasticity [51]. EMG has also been proposed to assess the physiological effects of the human–robot interaction [15, 52]. These outcome measures domains can describe the motor abilities previously identified. In particular, kinematics is able to quantify all motor abilities except for muscular effort and power, as already reported in the literature [3, 9, 35]. The electromyography, instead, could be exploited to assess efficiency, muscular effort, intra-limb coordination, planning predictability, power, and smoothness.

### Performance indicators

Considering the kinematics domain, we considered a set of PIs (Table 3) derived from the works of Nordin et al. [9], Garro et al. [3], de los Reyes-Guzmán et al. [16], and Schwarz et al. [35], which identified outcome measures suitable to describe the cause of impairment or correlated with clinical scales. With respect to the effort performed in these previous works towards standardization, we unified the PIs outlined in these works, deleted redundant PIs, and associated each PI with one of the *motor abilities* previously defined, as outlined in Table 3.

**Table 3:** Benchmarking indicators for motor abilities

Motor Ability	Performance Indicator	Domain		Mandatory/Recommended	Description	Relevant reference
		Kin	EMG			
Accuracy	Trajectory absolute error	x		R	Mean or maximal distance between ideal and actual trajectory between movement onset and end	[57]
	End-point error	x		R	Mean or maximal Euclidean distance between actual and target position at or after movement end	[58]
	Variable error	x		R	Standard deviation of the end-point error across multiple repetitions of the movement or task	[59]
	Area Index	x		R	Area between the desired straight line and the path actually performed	[60]
Efficacy	Success rate	x		R	Number of accomplished objectives (e.g., movements performed, tasks completed) divided by the total number of attempts	[61]
	Number of movements stops	x		M	Number of times that the velocity curve dropped below a percentage of peak velocity after movement onset	[62]
Efficiency	Movement time	x		R	Time from the onset to the end of a task or movement	[63]
	Path traveled	x		R	Path length covered between onset and end of a movement or task	[64]
	Path length ratio	x		M	Ratio between the path traveled and the shortest possible distance between movement onset and end	[65]
	Trunk compensation	x		R	Ratio between trunk displacement and hand displacement in the sagittal plane	[66]
	Waveform length		x	R	Cumulative length of the waveform of the EMG signal from the *i*^*th*^-muscle over the time segment	[67]
	Average amplitude change		x	R	Mean of the cumulative length of the waveform of the EMG signal from the *i*^*th*^-muscle over the time segment	[55]
	Difference absolute standard deviation value		x	R	Standard deviation of the cumulative length of the waveform of the EMG signal from the *i*^*th*^-muscle over the time segment	[55]
Intra-limb coordination	Joint angle correlation	x		R	Correlation between shoulder flexion–extension and elbow flexion–extension joint time angles profiles	[68]
	Elbow peak velocity	x		R	Highest value of the elbow flexion/extension joint velocity profile during movement	[43]
	Time to peak elbow extension angle	x		R	Time to reach peak extension angle for the elbow joint, relative to the duration of the movement	[69]
	Muscular synergies		x	R	Linear decomposition algorithm (e.g., principal components analysis, factor analysis, independent component analysis, and non-negative matrix factorization) to extract spatiotemporal, temporal, and spatial features from EMG signal of the muscles mainly involved in the task	[70]
	Co-contraction index		x	R	Percentage of overlapping activity of EMG linear envelopes between the agonist and the antagonist muscle involved in the task	[71]
	Intermuscular coherence		x	R	Square of the cross-spectra normalized with auto-spectra derived from the EMG signal from the agonist and the antagonist muscles involved in the task	[72]
Movement amplitude	Joint range of motion	x		M	Range of the anatomical joints angles between movement onset and end	[73]
	Maximum reached distance	x		R	Maximum distance reached from the starting position	[74]
	Trunk displacement	x		R	Euclidean distance covered by the trunk between movement onset and end	[75]
	Normalized reaching area	x		R	Maximally reached or reachable position during a movement or task divided by the length of the user’s arm	[76]
Muscular effort	Integrated EMG		x	R	Summation of rectified EMG signal amplitude obtained from the *ith*-muscle	[55]
	Root Mean Square		x	R	Square root of the mean square of the EMG signal amplitude obtained from the *ith*-muscle	[55]
	Activation level		x	M	Average of the absolute value of the EMG signal amplitude obtained from the *ith*-muscle in a segment	[77]
	Variance of EMG		x	R	Average of squared EMG signal amplitude obtained from the *ith*-muscle	[67]
	Mean absolute value slope		x	R	Differences between mean absolute values of the EMG signal amplitude obtained from the *ith*-muscle of the adjacent segments	[55]
Planning predictability	Time to peak velocity	x		R	Time to reach peak velocity relative to the duration of the movement	[78]
	Reaction time/Response latency	x		M	Time between the “Go” cue (as indicated by visual/acoustic feedback or any other channel) of a movement and the actual onset of the movement (e.g., 10% of peak velocity)	[62]
	Muscle onset		x	R	Time between the “Go” cue and the onset (e.g., detected by the Teager–Kaiser energy operator) of the EMG signal amplitude obtained from the *ith*-muscle	[79]
	Initial movement direction error	x		R	Distance between ideal and actual trajectory at an initial time point right after movement onset (e.g., 10% of peak velocity)	[62]
	Aiming angle	x		R	Angular difference between target direction and direction of travel calculated from starting point up to peak speed point	[80]
Power	Mean frequency		x	R	Sum of the product of the power spectrum of the EMG signal from the *ith*-muscle and the frequency divided by the total sum of the spectrum intensity	[56]
	Median frequency		x	R	Frequency at which the power spectrum of the EMG signal from the *i*^*th*^-muscle is divided into two regions with equal amplitude	[56]
	Mean power		x	R	Average power of the power spectrum of the EMG signal from the *i*^*th*^-muscle	[55]
	Power spectral density		x	R	Amount of power per frequency interval of the power spectrum of the EMG signal from the *ith*-muscle	[56]
	Frequency ratio		x	R	Ratio between the low-frequency components and the high-frequency components of the power spectrum of the EMG signal from the *ith*-muscle	[55]
	Power spectrum ratio		x	R	Ratio between the energy which is nearby the maximum value of the power spectrum of the EMG signal from the *ith*-muscle, and the energy which is the whole energy of the power spectrum	[55]
Smoothness	Number of velocity peaks	x		M	Number of peaks (i.e., maxima above a certain threshold) in the velocity profile between movement onset and end	[81]
	Speed correlation to idealized profile	x		R	Correlation between actual speed profile and idealized normal velocity profile (e.g., straight line)	[82]
	Movement Arrest Period Ratio	x		R	Proportion of time that movement speed exceeds a given percentage of peak speed	[83]
	Peak Speed Ratio	x		R	Mean speed divided by the peak speed	[83]
	Normalized dimensionless jerk	x		R	Time-integral of the squared jerk (i.e., third time-derivative of position) between movement onset and end normalized with respect to movement duration to the power of five and movement length to the power of two	[84]
	Spectral arc length	x		R	Length of the spectral trajectory (i.e., in the frequency domain) of the velocity profile between movement onset and end	[85]
	Mean acceleration	x		R	Mean value of the acceleration profile between movement onset and end	[86]
	EMG Zero Crossing		x	R	Number of times that amplitude values of the EMG signal from the *ith*-muscle crosses zero amplitude level	[55]
	Slope sign change		x	R	Number of times that slope of the EMG signal from the *ith*-muscle changes sign	[55]
Speed	Peak velocity	x		R	Maximal value of the velocity profile between movement onset and end	[87]
	Mean velocity	x		M	Mean value of the velocity profile between movement onset and end	[83]
	Mean velocity variability	x		R	Difference between the velocity profile of the participant’s reaching trajectory and the ideal velocity profile for each movement	[80]

If not specified, kinematic outcomes must be computed from a distal joint or the robot end-effector. Electromyography outcomes must be computed for every muscle recorded if not specified. Relevant reference specifies the first work of the literature that, to our knowledge, used that specific outcome

For the electromyography domain, instead, despite its huge potential, when evaluating movement or assessing the effect of the use of robots, researchers usually limit their analysis to standard outcomes (e.g., Root Mean Square, integrated EMG, co-contraction index) without a deep insight into the real meaning of these quantities and their relationship with motor abilities. Moreover, despite the recommendations [22], EMG measurements are not widely adopted in clinical settings [53, 54], and EMG signal features are more often used for control purposes than assessment ones. Therefore, we propose a list of PIs (Table 3) to assess the effects of rehabilitative interventions or for the control of upper limb devices [3, 55].

For each motor ability, we labeled as “mandatory” the PI that demonstrated the highest correlation with the Fugl-Meyer scale, and that does not need a normative reference value to be computed. In particular, for the kinematics domain, we relied on the review from Schwarz et al. [35], while for the electromyography domain, we based on the work of Cahyadi and colleagues [56]. For the motor abilities accuracy, intra-limb coordination, and power it was not possible to extract a mandatory PI because the literature lacks sufficient evidence of its correlation with the Fugl-Meyer scale.

### Benchmarking protocol

We proposed a worksheet designed to facilitate the execution and replication of the experiments (Table 4). The worksheet is constituted of three main sections: (1) definition of the system under investigation, (2) definition of experimental set-up and kinematics and electromyography standard definition, and (3) experimental procedure definition and standardization.

**Table 4:** Template of the worksheet to conduct the benchmarking

1) Definition of the system under investigation	
Subject Robotic device + Subject	
Subject description: Age: Sex: Pathology: Upper arm length (cm): Forearm length (cm): Dominant arm: Evaluated arm: Neuropsychological assessment:	Robotic device description (if present): Training modality: Number of DOFs: Actuated DOFs (list and level of resistance/assistance [− 1; 1]): Passive DOFs (list and level of antigravity compensation [0;1]):

2) Definition of experimental set-up and kinematics and electromyography standard definition	
Kinematics Sensor type: Sensors placement:	Electromyography Electrode types: Electrodes placement on muscles:

3) Experimental procedure definition and standardization	
3.1 Motor skills	Motor skills and number of repetitions: 1. Anterior reaching at rest position height (N =) 2. Anterior reaching at shoulder height (N =) 3. Moving objects at rest position height (N =) 4. Moving objects at shoulder height (N =) 5. Hand to mouth (N =)
	Environment: Micro-gravity Gravity
	Description of the object (if skill number 2 or 3): Shape: Dimensions:
3.2 Disturbance	Description of the disturbance Examples: Payload Description (mass in kg): Cognitive disturbance Description: Motor perturbation Description (Direction, location, magnitude, frequency, cycle waveform): Other Description:
3.3 Outcomes	Outcome measures categories: Kinematics Electromyography
3.4 Protocol	Setting: The subject sits in front of a desk on a chair without an armrest and with the seatback blocked at 90° The height of the desk is adjusted in order to have the elbow at 90° of flexion and no compensation of the shoulder in the frontal plane The starting position is with the hand on the desk in a central position (rest position) The trunk of the subject must be blocked. Trunk displacement must be recorded, and if higher than 20° in the sagittal or frontal plane, the test is not valid Procedure: 1) Set the measurement system according to the required outcome variables 2) Ask the subject to sit as described in section “Setting” 3) If skill 2 or 3, locate the object in the target position 4) (Eventually, set disturbance) 5) Start trial (data saving start) 6) Stop trial (data saving stop) 7) Store recorded data 8) Analyze data according to selected Outcomes

### Definition of the system under investigation

**Fig. 3:**
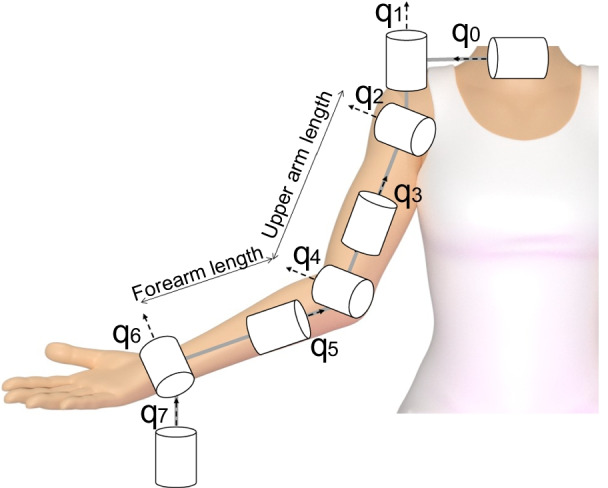
Upper limb kinematics model according to robotics convention

First, the user must select if the protocol will be conducted on a subject alone or an end-user wearing a robotic device. The worksheet includes a brief description of the subject. In particular, the following data are required for correct identification of normative reference data: age, sex, pathology, upper arm and forearm lengths, neuropsychological assessment, dominant and evaluated arm. The device, if included, has to be characterized in terms of (1) device type (i.e., exoskeleton, end-effector, soft device), (2) training/assistive modality, (3) number of degrees of freedom (DOFs), (4) details on number and list of actuated and passive DOFs. For the training modality, we suggest the classification proposed by Basteris and colleagues [88], which proposed eight different modes that characterize the human and robot’s contribution during the execution of the motor skills. For each active DOF, it is necessary to specify the list of human joints (Fig. 3), and the level of robot contribution. In particular, with active DOF, the rater must quantify the level of resistance/assistance in the normalized range [− 1; + 1]. − 1 corresponds to the “resistive” modality with a resistive level to counterbalance the maximum voluntary contraction of the user against that DOF, while + 1 is the “robot-in-charge” training modality (i.e., the movement is performed by the robot regardless of the subject’s response [88]). The “transparent mode” (i.e., “the robot does not provide assistance, nor resistance to the movement [88, 89]”) corresponds to 0. For passive DOFs, instead, the rater must specify the level of gravity compensation, ranging from 0 (i.e., the robot is not compensating for gravity—“transparent” modality) to 1 (i.e., the robot compensates for the weight of the user’s arm completely).

### Experimental set-up

**Fig. 4:**
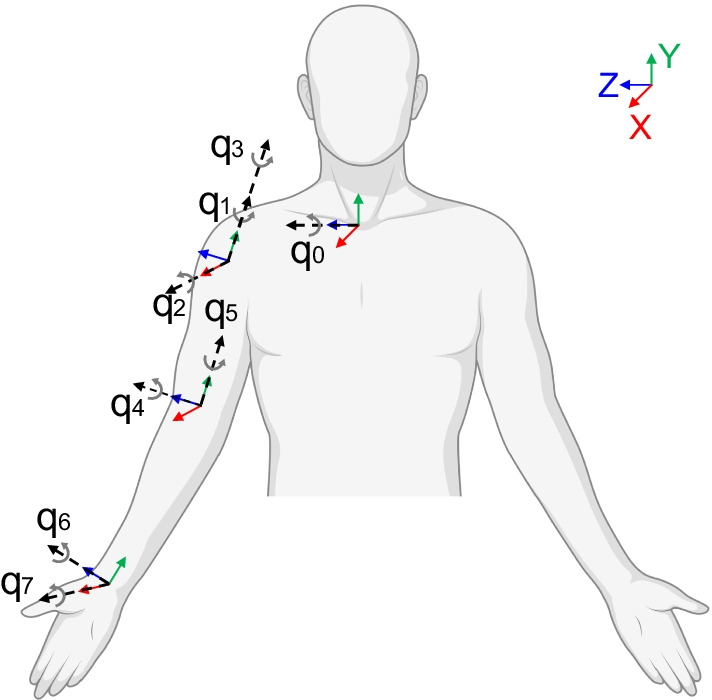
Upper limb kinematics model according to ISB guidelines

In Sect. 2, the user has to describe the instrumented experimental set-up. For kinematics, the specifications concern the type of sensor and their positioning on the anatomical segments. For electromyography, instead, the user must specify the selected muscles and the electrodes type (e.g., wired/wireless, superficial/intramuscular). We proposed a standardized kinematics model to calculate all the PIs in Table 3 properly, and we identified the most relevant upper limb muscles involved in the motor skills of the scheme. Considering kinematics, an accurate description of the human upper limb is challenging due to the high complexity of its structure [90]. In this framework, pursuing the objective of feasibility, a trade-off between complexity and accuracy is necessary. Therefore, we suggest the model presented by [91], adapting it to respect the recommendations of the International Society of Biomechanics (ISB) [92] (Fig. 4). In particular, the thorax is represented by a single DOF corresponding to the flexion/extension (q_0_). The shoulder is simplified as a ball-and-socket joint represented by the glenohumeral joint. Indeed, the shoulder motion can be represented largely by the glenohumeral joint for a variety of arm activities involving up to 90° of arm elevation [91], which is our case. The corresponding three DOFs are plane of elevation (q_1_), elevation angle (q_2_), and axial rotation (q_3_). Two DOFs can represent the elbow: flexion/extension (q_4_) and pronation/supination (i.e., axial rotation of the forearm—q_5_). Finally, the wrist is characterized by two DOFs: flexion/extension (q_6_) and ulnar/radial deviation (q_7_). Considering the shoulder joint, often researchers in the robotics field use a different convention, represented by these angles: flexion/extension, horizontal adduction/abduction, and humeral rotation (Fig. 3). The kinematic transformations between frames are presented in [93]. The PIs listed in Table 3 concern both measures at the single joint (e.g., joint angle correlation), and at the end-effector level (e.g., end-point error). The proposed kinematics model is required to correctly compute all the PIs.

**Fig. 5:**
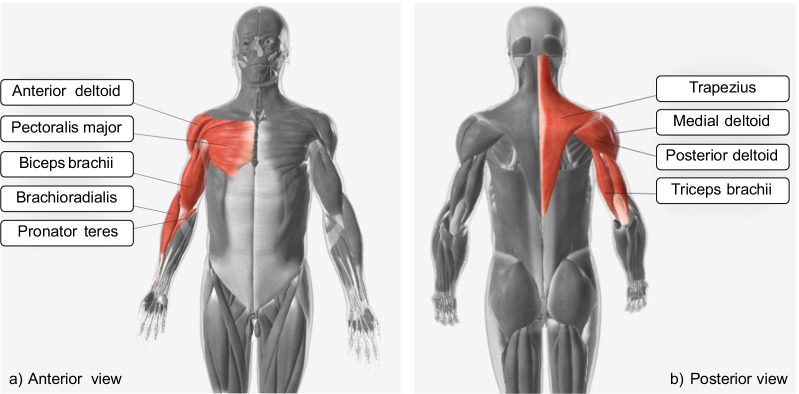
Upper limb main muscles involved in motor skills defined in the benchmarking scheme

In what relates to the electromyography, we identified the following muscles as the most relevant to the motor skills we proposed: trapezius descendens, pectoralis major, anterior deltoid, medial deltoid, posterior deltoid, triceps brachii (long head), biceps brachii (long head), brachioradialis, and pronator teres (Fig. 5). Sensor placement, signal processing, and modeling should follow the SENIAM (Surface ElectroMyoGraphy for the Non-Invasive Assessment of Muscles) guidelines [94].

### Experimental procedure

**Fig. 6:**
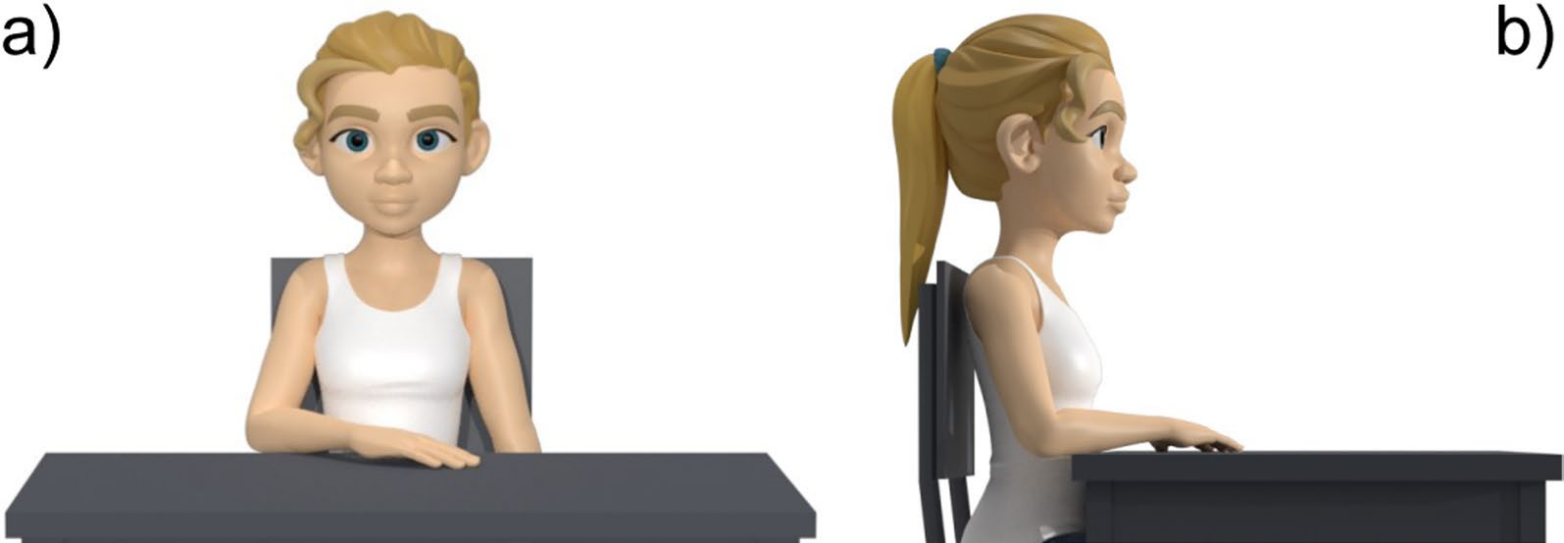
Rest position (A) in the frontal view (**a**) and in the lateral view (**b**)

**Fig. 7:**
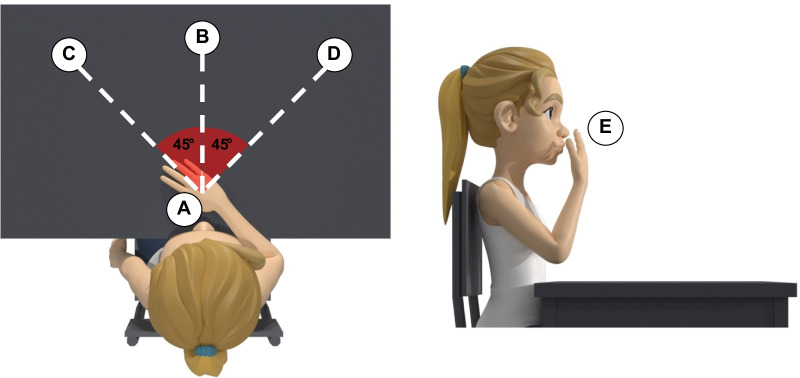
Target points or object location for motor skill anterior reaching and move object. **A** = Rest position; **B** = Central position; **C** = Controlateral position; **D** = Ipsilateral position; **E** = Mouth

The third part is related to the definition of the experimental procedure. The first section describes the motor skills, the environment, and the description of the (possible) object. For all the motor skills, the subject is seated in front of a desk on a chair without an armrest and with the seatback blocked with a tilt angle between 100° and 110°. The starting position is with the hand on the desk in a comfortable position, with the palm down and with the center of the palm of the hand aligned with the user’s navel (A—rest position) (Fig. 6). The height of the desk should be adjusted to have the elbow at 90° of flexion and no compensation of the shoulder in the frontal plane when the subject has the arm in the rest position. If the patient cannot reach this position autonomously, the rater can passively position the patient’s arm in the starting position. As to the target points, in the *anterior reaching* and *move objects* motor skills, they can be placed at two different heights, according to the assessor’s choice: at the same height of the rest position or the subject’s shoulder height. The rest point, instead, does not change. Consequently, these motor skills are split into (1) *anterior reaching at rest position height*, (2) *anterior reaching at shoulder height*, (3) *move objects at rest position height*, and (4) *move objects at shoulder height*. The subject has to carry out the movements without moving his/her back away from the backrest to avoid compensation with the trunk. Movements are performed at a self-selected speed. During the *anterior reaching* motor skills, both at rest position height and at shoulder height, starting from the rest position (A), the subject has to reach three target points placed in the central (B), contralateral (C), and ipsilateral positions (D) (Fig. 7). After each reach, the subject must return to the rest position (A) (Table 5). Point B is placed in front of the subject and aligned with point A. Points C and D are located at 45 degrees with respect to the straight line connecting point A with point B (Fig. 7). The three target points (B, C, and D) must be placed at the distance corresponding to a complete elbow extension of the subject’s arm in that direction. In the *moving objects* motor skills, the starting position is the rest position (A), and the object is placed in the central position (B). The subject must grasp the object in the central position (B), then push/pull it to reach two target positions at contralateral (C) and ipsilateral (D) (Fig. 7). After each reaching, the subject must release the object and return to the starting position (A). Lastly, the object will be returned to the initial central position (B) (Table 5). Finally, the *hand to mouth* motor skill is subdivided into two cases: without and with the object. In the first case, starting with the hand on the desk in the rest position (A), the subject is asked to reach his/her mouth (E) and touch it with the palm. After the idle phase, the subject has to return to the rest position (A). Instead, the case with the object consists of the activity of daily living mimicking the drinking task. Starting with the hand on the desk in the rest position (A), the subject has to grasp an object close to the rest position (A), reach his/her mouth (E) with the hand and the object, return to the start position on the plane (A), then release the object and position his/her hand in the rest configuration (Table 5). During this task, the subject is asked not to move the head toward the hand.

**Table 5:** Motor skills flow description through motor primitives

Motor skill	1) Anterior reaching at rest position height 2) Anterior reaching at shoulder height	3) Move objects at rest position height 4) Move objects at shoulder height	5) Hand to mouth without object	6) Hand to mouth with object
Motor primitives	1) Idle (A) 2) Point-to-point reach (B) 3) Reposition (A) 4) Point-to-point reach (C) 5) Reposition (A) 6) Point-to-point reach (D) 7) Reposition (A)	1) Idle (A) 2) Reach for grasp (B) 3) Transport (C) 4) Reposition (A) 5) Idle (A) 6) Reach for grasp (C) 7) Transport (D) 8) Reposition (A) 9) Idle (A) 10) Reach for grasp (D) 11) Transport (B) 12) Reposition (A)	1) Idle (A) 2) Point-to-point reach (E) 3) Idle (E) 4) Reposition (A)	1) Idle (A) 2) Reach for grasp (A) 3) Transport (E) 4) Stabilize (E) 5) Transport (A) 6) Reposition (A)

If the protocol is executed with a robotic device, the environment will be classified as *gravity* if the training modality is “patient-in-charge”, “transparent”, or “resistive”. Otherwise, the environment will be *microgravity*, in order to take into account the assistance and gravity compensation provided by the robot.

The motor skill *anterior reaching at rest position height* represents the easiest movement that can be analyzed, and it is suitable for patients unable to grasp objects or elevate their arm against gravity. This motor skill, together with the motor skill *move objects at rest position height,* can be performed by sliding the arm on the table (hence, in the *micro-gravity* environment).

The motor skills *moving objects* and *hand to mouth with object* involve the mobilization of an object. In order to build a standardized and replicable benchmarking scheme, the object is represented by a cylindrical object of daily life (i.e., 0.5 l empty water bottle).

We suggest at least eight repetitions for each motor skill as a compromise between data robustness and repeatability, and the time required for the protocol.

The following part of the worksheet (i.e., disturbances) has to be filled only if disturbances are present during the experiment. The experimenter sets the disturbance. A possible disturbance is represented by a payload in the object (e.g., a bottle filled with water). Other disturbances (e.g., cognitive disturbance, motor perturbation) have to be carefully characterized.

The last part of the worksheet drives the assessor to execute the protocol. Before the execution of the protocol, the rater must explain the movements accurately to the subject. During the examination, verbal cues and encouragement must be avoided. In this way, the obtained output is only due to the patient’s performance and abilities. Moreover, verbal stimuli are difficult to standardize and reproduce.

## Discussion

Technologies and sensors, such as optoelectronic systems, inertial measurement units, or EMG devices, can provide valid, reliable, and sensitive assessment tools exploitable in neurorehabilitation to objectively investigate sensorimotor impairments. Moreover, recently, some robotic devices, such as exoskeletons, are demonstrating their potential to be used not only as a complement to conventional therapy but also to assess sensorimotor capabilities in a more objective way and under repeatable conditions [13, 14]. There is now a clear need for guidelines for clinicians and researchers to optimize technology-based assessment since standardized international evidence-based guidelines are missing, especially considering the upper limb district [22, 27, 28].

This work defines a unified scheme for benchmarking upper limb capabilities that can be used in the neurorehabilitation field in several ways. In the acute phase, this assessment procedure can be used to evaluate the level of motor impairment and personalize the intervention according to the patient’s needs. In subsequent phases, the scheme can be exploited for tuning training parameters (e.g., type and complexity of a task, required amount of body weight support, percentage of active assistance) to adapt and optimize the level of challenge during rehabilitation. After the end of an intervention, the scheme could be exploited to assess eventual patient capabilities’ improvements.

Traditionally, assessment procedures in neurorehabilitation are based on standard clinical scales, selected among the International Classification of Functioning (ICF), Disability and Health domains [95] (e.g., Fugl-Meyer Assessment, Action Research Arm Test). In rehabilitation medicine, these scales represent the fundamental basis of the so-called Evidence-Based Medicine, which is defined as the best available evidence in the process of decision-making related to patients’ health care [96]. However, relying only on clinical scales could not be sufficient to provide an accurate evaluation, as already pointed out by the scientific community [7, 22, 28, 97]. Therefore, we suggest integrating traditional Evidence-Based Medicine with the proposed benchmarking scheme. In particular, following the definition of the World Health Organization, this scheme could be exploited as a “Capacity qualifier” [98]. Indeed, it describes an individual’s ability to execute a task or an action without considering the environment, which can be considered irrelevant being a standardized evaluation setting. This scheme could increase the relevance and accuracy of the assessment. Indeed, it allows valuable comparisons, both considering patients’ longitudinal evaluation at different time points or the comparison of the efficacy of different rehabilitative interventions. The scheme applied to users without external devices could enable data comparison across clinical and research trials, possibly leading to more robust and shared evidence. At the same time, quantitative outcome measures are characterized by higher precision, finer rate, and repeatability.

The motor skills constituting the protocol are suitable for a clinically relevant evaluation, for different levels of abilities, and can be easily decomposed in motor primitives. These simple motor primitives are determinants of Capacity, and they could be used to determine the cause of the eventual impairment. Moreover, they could be exploited to derive or predict the performance of more complex skills without the need for a benchmark tailored for all possible upper limb movements. We included a minimal experimental set-up that is easy to administer and is currently present in most clinical settings. Although other aspects might be relevant in evaluating upper limb capabilities, such as kinetic evaluation, we decided to include kinematics and electromyography domains, which can also be assessed without robots. As suggested by Torricelli and colleagues [19], the scheme should be designed to maximize its transferability across different scenarios and subjects. Indeed, it leaves a certain degree of freedom in the benchmarking protocol, and, in this way, it can be implemented on various robotic platforms or adapted to different laboratory equipment. Consequently, the applicability of this benchmarking could be broad, and it may be considered an important tool for routinely upper limb rehabilitative technologies functional evaluation, leading to platform-independent assessments that can allow the comparison of treatment outcomes across rehabilitation centers worldwide.

The benchmarking scheme could also be exploited to assess the impact of a robot on the user’s performance, by comparing the subject’s performance without and with the external device. Indeed, the presence of the robot, as well as different levels of assistance/resistance, influence the PIs. The application of the scheme could allow quantifying both the effect of the robot and of different training modalities, comparing them with the baseline performance of the user without any external device. In this view, the scheme could also be exploited to assess the effectiveness of assistive arm supports.

Although this framework is meant especially for disorders that occur in terms of weakness or hemiparesis (e.g., stroke), the scheme could be easily extended to other neurological conditions (e.g., cerebral palsy). Indeed, the involved motor skills are based on the decomposition into motor primitives of daily life activities that are relevant for each pathology. As a consequence, the motor abilities and performance indicators can constitute a common reference landscape. The performance indicators must be interpreted in relation to the pathology, the site of injury, or the related clinical conditions specific to the patient.

In line with Torricelli et al. [19], a benchmarking framework should fulfill the following basic requirements: feasibility, reproducibility, and transferability. The feasibility can be defined as the capability of the scheme to be successfully used in the given application field, i.e., the clinical setting in our case [99]. Reproducibility is defined as “the obtention of comparable results by different teams, measuring systems, and locations” [100]. Transferability is defined as “the ability to predict how a system would behave in the real world, by means of experiments conducted in a controlled (typically laboratory) environment” [15]. Finally, the scheme has to be clinically meaningful, i.e., it has to constitute a relevant decision-making support system for clinicians in the neurorehabilitation context.

We designed this benchmarking scheme to respect these four requirements. However, the effective compliance of the scheme with such requirements needs to be demonstrated experimentally. Indeed, this work represents the starting point for creating a consensus among the scientific community. An iterative process involving stakeholders or players in the rehabilitation fields (e.g., physicians, therapists, engineers) is necessary to obtain a definitive consensus on this scheme.

A possible plan in this direction could include a test–retest analysis on a population of healthy subjects to validate the feasibility and reproducibility of the scheme also in terms of inter-rater reproducibility. In this way, the normative data necessary to compute the baseline required by some indicators (e.g., the optimal trajectory) can also be derived. Finally, the reproducibility should be verified across different instrumentation (e.g., performing the benchmarking scheme with optoelectronic systems and inertial measurement units for the kinematics). To validate the transferability, it is necessary to investigate the correlation and agreement between the PIs obtained by this scheme and standard clinical scales that evaluate daily life movements (i.e., ICF Activity and Participation domains) or questionnaires assessing the quality of life in a real-life environment. Finally, to validate the clinical meaningfulness, the results from this framework need to be correlated with those from standard clinical scales among a population of neurological patients.

Specific updates of the benchmarking scheme might be proposed to assure the compliance of the scheme with the stated requirements after tests in the relevant environment. Moreover, the application of the scheme could lead to an accurate description of human upper limb movements, which could be useful in the neurorehabilitation field in different ways. It can support the path planning process during robot development (e.g., as a combination of primitives), the recognition of pathological movements through artificial intelligence algorithm, or it could be beneficial to improve the human-likeness of robots.

In this work, we decided to propose a framework for the rehabilitative scenario without losing generalizability. This benchmark can be translated to different healthcare domains with proper customization. For example, it can be adopted to evaluate the effectiveness of assistive devices to support daily life activities in subjects with neurodegenerative diseases, analyzing the subject’s performance during the execution of the protocol without and with arm support. In this case, the PIs should be chosen among those related to task accomplishment (e.g., success rate, active movement index, movement time, joint range of motion). Moreover, our scheme may be exploited to evaluate bimanual tasks or interventions done with bimanual exoskeletons, adding proper measures on inter-limb coordination, as suggested by Nordin and colleagues [9]. The identified motor primitives and most of the PIs could also be transferred to the case of occupational exoskeleton for the evaluation of physiological changes induced by the exoskeleton (e.g., variations in the Root Mean Square of EMG signal or in joint range of motion). Nevertheless, the protocol should be carefully revised to be adapted to the specific application.

Despite the relevance of this work, some limitations can be identified. First, this benchmarking scheme is intended to evaluate only the upper limb (i.e., shoulder, elbow, and wrist) and not the hand. Although the upper limb and the hand synergistically provide integrated functions, from the point of view of rehabilitation protocols, clinical assessment, and diagnosis, they represent different districts. Standard clinical scales (e.g., the Action Research Arm Test) tackle arm and hand with different items, and most of the existing robots for rehabilitation are designed for the arm only (e.g., ArmeoPower by Hocoma, Harmony by Harmonic Bionics) or the hand only (Gloreha by Idrogenet, Hand of Hope by Rehab-Robotics). In line with the idea of considering hand and arm evaluation as separate, an instrumented assessment tool has already been proposed [101]. Considering the outcome measures, in this scheme, we focused only on PIs achievable from sensors. A comprehensive evaluation of technologies should also include the user experience, including perceptual, emotional, and cognitive aspects [15]. For upper limb assistive technologies, for example, it was demonstrated that subject’s self-perceived improvement was significantly greater than the functional gain detectable through clinical scales or a system measurement [102]. Another important aspect we did not consider was the physical human–robot interaction, which includes the kinematic compatibility and interaction forces/torques between the system and the subject’s joints and ergonomics evaluations. The scheme does not include bimanual tasks. Although relevant in the context of neurological disorders, bimanual tasks are usually related to the grasping of objects and, hence, to hand functions, which is out of the scope of this benchmarking scheme. At the same time, a requirement of the scheme is its feasibility in the context of robotic devices (e.g., exoskeletons), which are in great majority unilateral. Moreover, the clinical evaluation of post-stroke people, which represent a main cause of disability worldwide, is unilateral. Finally, other relevant aspects, such as tremor, are not addressed in this scheme and would need a revised version.

## Conclusion and future perspectives

Benchmarking represents the desirable approach for evaluating the upper limb abilities of frail subjects and assessing and comparing the performance of different rehabilitative interventions. In this context, technology-driven solutions provide a promising complement to conventional clinical assessments. We created a benchmarking framework based on kinematics and electromyography domains to evaluate the upper limb capabilities. The scheme can be exploited to assess the effectiveness of a rehabilitative program, e.g., comparing patients’ performance before and after the intervention, or to perform an instrumented clinical evaluation of a patient. It is suitable to be conducted with robot-equipped sensors as well as with external sensors (e.g., optoelectronic system, wearable sensors). We suggest that this framework should be combined with the standard Evidence-Based Medicine relying only on clinical scales. The scheme could serve as a complementary and objective tool that promises to reveal sensorimotor impairment profiles more accurately, potentially allowing for a reduction of the required sample size for clinical trials.

Future efforts are needed to validate the reproducibility, transferability, and clinical meaningfulness of the scheme and eventually revise it. This scheme aims to be largely used by the scientific community to create a shared database of human performance that could drive the development of new personalized technologies.

## Data Availability

Not applicable.

## Abbreviations

EMG: Electromyography
PI: Performance indicator
DOF: Degree of freedom
ISB: International Society of Biomechanics
SENIAM: Surface ElectroMyoGraphy for the Non-Invasive Assessment of Muscles

## References

[1] Schwarz A, Bhagubai MMC, Nies SHG, Held JPO, Veltink PH, Buurke JH (2022). Characterization of stroke-related upper limb motor impairments across various upper limb activities by use of kinematic core set measures. J NeuroEngineering Rehabil.

[2] Lambercy O, Lünenburger L, Gassert R, Bolliger M, Dietz V, Nef T, Rymer WZ (2012). Robots for Measurement/Clinical Assessment. Neurorehabilitation Technology [Internet].

[3] Garro F, Chiappalone M, Buccelli S, Michieli LD, Semprini M (2021). Neuromechanical biomarkers for robotic neurorehabilitation. Front Neurorobot [Internet]..

[4] Rodgers H, Bosomworth H, Krebs HI, van Wijck F, Howel D, Wilson N (2019). Robot assisted training for the upper limb after stroke (RATULS): a multicentre randomised controlled trial. The Lancet.

[5] Mehrholz J, Pohl M, Platz T, Kugler J, Elsner B (2018). Electromechanical and robot-assisted arm training for improving activities of daily living, arm function, and arm muscle strength after stroke. Cochrane Database Syst Rev.

[6] Scott SH, Dukelow SP (2011). Potential of robots as next-generation technology for clinical assessment of neurological disorders and upper-limb therapy. J Rehabil Res Dev.

[7] Schwarz A, Averta G, Veerbeek JM, Luft AR, Held JPO, Valenza G (2019). A functional analysis-based approach to quantify upper limb impairment level in chronic stroke patients: a pilot study. Annu Int Conf IEEE Eng Med Biol Soc.

[8] Thrane G, Sunnerhagen KS, Persson HC, Opheim A, Alt MM (2019). Kinematic upper extremity performance in people with near or fully recovered sensorimotor function after stroke. Physiother Theory Pract.

[9] Nordin N, Xie SQ, Wünsche B (2014). Assessment of movement quality in robot- assisted upper limb rehabilitation after stroke: a review. J Neuroeng Rehabil.

[10] Aller F, Pinto-Fernandez D, Torricelli D, Pons JL, Mombaur K (2020). From the state of the art of assessment metrics toward novel concepts for humanoid robot locomotion benchmarking. IEEE Robot Automation Lett.

[11] Grimm F, Kraugmann J, Naros G, Gharabaghi A (2021). Clinical validation of kinematic assessments of post-stroke upper limb movements with a multi-joint arm exoskeleton. J Neuroeng Rehabil.

[12] Belfatto A, Scano A, Chiavenna A, Mastropietro A, Mrakic-Sposta S, Pittaccio S (2018). A multiparameter approach to evaluate post-stroke patients: an application on robotic rehabilitation. Appl Sci.

[13] De Oliveira AC, Sulzer JS, Deshpande AD (2021). Assessment of upper-extremity joint angles using harmony exoskeleton. IEEE Trans Neural Syst Rehabil Eng.

[14] Pasquini M, James ND, Dewany I, Coen FV, Cho N, Lai S (2022). Preclinical upper limb neurorobotic platform to assess, rehabilitate, and develop therapies. Sci Robot.

[15] Torricelli D, Rodriguez-Guerrero C, Veneman JF, Crea S, Briem K, Lenggenhager B (2020). Benchmarking wearable robots: challenges and recommendations from functional, user experience, and methodological perspectives. Front Robot AI.

[16] de los Reyes-Guzmán A, Dimbwadyo-Terrer I, Trincado-Alonso F, Monasterio-Huelin F, Torricelli D, Gil-Agudo A (2014). Quantitative assessment based on kinematic measures of functional impairments during upper extremity movements: a review. Clin Biomech (Bristol, Avon).

[17] Tran VD, Dario P, Mazzoleni S (2018). Kinematic measures for upper limb robot-assisted therapy following stroke and correlations with clinical outcome measures: a review. Med Eng Phys.

[18] Scotto di Luzio F, Cordella F, Lauretti C, Draicchio F, Zollo L, Masia L, Micera S, Akay M, Pons JL (2019). Assessment of muscular activation patterns in 3D upper limb robot-aided rehabilitation. Converging clinical and engineering research on neurorehabilitation III.

[19] Torricelli D, Gonzalez-Vargas J, Veneman JF, Mombaur K, Tsagarakis N, del Ama AJ (2015). Benchmarking bipedal locomotion: a unified scheme for humanoids, wearable robots, and humans. IEEE Robot Automation Magazine..

[20] Definition of BENCHMARK [Internet]. [cited 2022 Feb 9]. Available from: https://www.merriam-webster.com/dictionary/benchmark.

[21] Torricelli D, Pons JL, Carrozza MC, Micera S, Pons JL (2019). EUROBENCH: preparing robots for the real world. Wearable robotics: challenges and trends.

[22] Hughes AM, Bouças SB, Burridge JH, Alt Murphy M, Buurke J, Feys P (2016). Evaluation of upper extremity neurorehabilitation using technology: a European Delphi consensus study within the EU COST Action Network on Robotics for Neurorehabilitation. J Neuroeng Rehabil.

[23] Bayón C, Delgado-Oleas G, Avellar L, Bentivoglio F, Di Tommaso F, Tagliamonte NL (2022). Development and evaluation of benchbalance: a system for benchmarking balance capabilities of wearable robots and their users. Sensors.

[24] Dacal-Nieto A, Masood J, Vergara D, Alves M, Torricelli D, Akay M, Pons JL (2022). TestEd information system: automatic evaluation of exoskeletons subjective performance and user experience. Converging clinical and engineering research on neurorehabilitation IV.

[25] Maugliani N, Caimmi M, Malosio M, Airoldi F, Borro D, Rosquete D, Moreno JC, Masood J, Schneider U, Maufroy C, Pons JL (2022). Lower-limbs exoskeletons benchmark exploiting a stairs-based testbed: the STEPbySTEP Project. Wearable robotics: challenges and trends.

[26] Taborri J, Salvatori S, Mariani G, Rossi S, Patanè F. BEAT: Balance Evaluation Automated Testbed for the standardization of balance assessment in human wearing exoskeleton. In: 2020 IEEE International Workshop on Metrology for Industry 40 IoT. 2020. p. 526–31.

[27] Burridge J, Alt Murphy M, Buurke J, Feys P, Keller T, Klamroth-Marganska V (2019). A systematic review of international clinical guidelines for rehabilitation of people with neurological conditions: what recommendations are made for upper limb assessment?. Front Neurol.

[28] Prange-Lasonder GB, Alt Murphy M, Lamers I, Hughes AM, Buurke JH, Feys P (2021). European evidence-based recommendations for clinical assessment of upper limb in neurorehabilitation (CAULIN): data synthesis from systematic reviews, clinical practice guidelines and expert consensus. J NeuroEngineering Rehabil.

[29] Schambra HM, Parnandi A, Pandit NG, Uddin J, Wirtanen A, Nilsen DM (2019). A taxonomy of functional upper extremity motion. Front Neurol [Internet]..

[30] Magill RA, Anderson D (2014). Motor learning and control: concepts and applications.

[31] Gentile AM. Skill acquisition: action, movement, and neuromotor processes. Movement Science: Foundations for Physical Therapy. 2000;93–154.

[32] Guerra J, Uddin J, Nilsen D, Mclnerney J, Fadoo A, Omofuma IB, et al. Capture, learning, and classification of upper extremity movement primitives in healthy controls and stroke patients. In: 2017 International Conference on Rehabilitation Robotics (ICORR). 2017. p. 547–54. 10.1109/ICORR.2017.8009305 PMC5645799 28813877

[33] Pissadaki EK, Abrami AGS, Heisig SJ, Bilal E, Cavallo M, Wacnik PW (2018). Decomposition of complex movements into primitives for Parkinson’s disease assessment. IBM J Res Dev.

[34] Miranda JG, Daneault JF, Vergara-Diaz G, Quixadá AP, Fonseca MD (2018). Complex upper-limb movements are generated by combining motor primitives that scale with the movement size. Sci Rep.

[35] Schwarz A, Kanzler CM, Lambercy O, Luft AR, Veerbeek JM (2019). Systematic review on kinematic assessments of upper limb movements after stroke. Stroke.

[36] Birkenmeier RL, Prager EM, Lang CE (2010). Translating animal doses of task-specific training to people with chronic stroke in 1-hour therapy sessions: a proof-of-concept study. Neurorehabil Neural Repair.

[37] Caimmi M, Guanziroli E, Malosio M, Pedrocchi N, Vicentini F, Molinari Tosatti L (2015). Normative data for an instrumental assessment of the upper-limb functionality. Biomed Res Int.

[38] Lang CE, Bland MD, Bailey RR, Schaefer SY, Birkenmeier RL (2013). Assessment of upper extremity impairment, function, and activity after stroke: foundations for clinical decision making. J Hand Therapy..

[39] Armbrüster C, Spijkers W (2006). Movement planning in prehension: do intended actions influence the initial reach and grasp movement?. Mot Control.

[40] Becchio C, Manera V, Sartori L, Cavallo A, Castiello U (2012). Grasping intentions: from thought experiments to empirical evidence. Front Human Neurosci [Internet]..

[41] Ambrosini E, Zajc J, Ferrante S, Ferrigno G, Gasperina SD, Bulgheroni M (2019). A hybrid robotic system for arm training of stroke survivors: concept and first evaluation. IEEE Trans Biomed Eng.

[42] Puchinger M, Kurup NBR, Keck T, Zajc J, Russold MF, Gföhler M. The Retrainer Light-Weight Arm Exoskeleton: Effect of Adjustable Gravity Compensation on Muscle Activations and Forces. In: 2018 7th IEEE International Conference on Biomedical Robotics and Biomechatronics (Biorob). 2018. p. 396–401.

[43] Alt Murphy M, Willén C, Sunnerhagen KS (2011). Kinematic variables quantifying upper-extremity performance after stroke during reaching and drinking from a glass. Neurorehabil Neural Repair.

[44] Caimmi M, Carda S, Giovanzana C, Maini ES, Sabatini AM, Smania N (2008). Using kinematic analysis to evaluate constraint-induced movement therapy in chronic stroke patients. Neurorehabil Neural Repair.

[45] Carpinella I, Lencioni T, Bowman T, Bertoni R, Turolla A, Ferrarin M (2020). Effects of robot therapy on upper body kinematics and arm function in persons post stroke: a pilot randomized controlled trial. J Neuroeng Rehabil.

[46] Goffredo M, Mazzoleni S, Gison A, Infarinato F, Pournajaf S, Galafate D (2019). Kinematic parameters for tracking patient progress during upper limb robot-assisted rehabilitation: an observational study on subacute stroke subjects. Appl Bionics Biomech.

[47] Besomi M, Hodges PW, Clancy EA, Van Dieën J, Hug F, Lowery M (2020). Consensus for experimental design in electromyography (CEDE) project: amplitude normalization matrix. J Electromyogr Kinesiol.

[48] Mugnosso M, Marini F, Holmes M, Morasso P, Zenzeri J (2018). Muscle fatigue assessment during robot-mediated movements. J Neuroeng Rehabil.

[49] Lencioni T, Fornia L, Bowman T, Marzegan A, Caronni A, Turolla A (2021). A randomized controlled trial on the effects induced by robot-assisted and usual-care rehabilitation on upper limb muscle synergies in post-stroke subjects. Sci Rep.

[50] Nam C, Rong W, Li W, Xie Y, Hu X, Zheng Y (2017). The effects of upper-limb training assisted with an electromyography-driven neuromuscular electrical stimulation robotic hand on chronic stroke. Front Neurol [Internet]..

[51] Longatelli V, Pedrocchi A, Guanziroli E, Molteni F, Gandolla M (2021). Robotic exoskeleton gait training in stroke: an electromyography-based evaluation. Front Neurorobot [Internet]..

[52] Bi L, Feleke AG, Guan C (2019). A review on EMG-based motor intention prediction of continuous human upper limb motion for human-robot collaboration. Biomed Signal Process Control.

[53] Campanini I, Disselhorst-Klug C, Rymer WZ, Merletti R (2020). Surface EMG in clinical assessment and neurorehabilitation: barriers limiting its use. Front Neurol.

[54] Manca A, Cereatti A, Bar-On L, Botter A, Della Croce U, Knaflitz M (2020). A survey on the use and barriers of surface electromyography in neurorehabilitation. Front Neurol.

[55] Phinyomark A, Phukpattaranont P, Limsakul C (2012). Feature reduction and selection for EMG signal classification. Expert Syst Appl.

[56] Cahyadi BN, Khairunizam W, Muhammad MN, Zunaidi I, Majid SH, Rudzuan MN, et al. Analysis of EMG based Arm Movement Sequence using Mean and Median Frequency. In: 2018 5th International Conference on Electrical Engineering, Computer Science and Informatics (EECSI). 2018. p. 440–4.

[57] Colombo R, Pisano F, Micera S, Mazzone A, Delconte C, Carrozza MC (2005). Robotic techniques for upper limb evaluation and rehabilitation of stroke patients. IEEE Trans Neural Syst Rehabil Eng.

[58] Casadio M, Morasso P, Noriaki Ide A, Sanguineti V, Giannoni P (2009). Measuring functional recovery of hemiparetic subjects during gentle robot therapy. Measurement.

[59] Patterson TS, Bishop MD, McGuirk TE, Sethi A, Richards LG (2011). Reliability of upper extremity kinematics while performing different tasks in individuals with stroke. J Mot Behav.

[60] Tropea P, Cesqui B, Monaco V, Aliboni S, Posteraro F, Micera S (2013). Effects of the alternate combination of “error-enhancing” and “active assistive” robot-mediated treatments on stroke patients. IEEE J Transl Eng Health Med.

[61] Piron L, Tonin P, Cortese F, Zampolini M, Piccione F, Agostini M, et al. Post-stroke arm motor telerehabilitation web-based. In: 2006 International Workshop on Virtual Rehabilitation. 2006. p. 145–8.

[62] Coderre AM, Zeid AA, Dukelow SP, Demmer MJ, Moore KD, Demers MJ (2010). Assessment of upper-limb sensorimotor function of subacute stroke patients using visually guided reaching. Neurorehabil Neural Repair.

[63] Adamovich S, Fluet GG, Merians AS, Mathai A, Qiu Q. Recovery of hand function in virtual reality: Training hemiparetic hand and arm together or separately. In: 2008 30th Annual International Conference of the IEEE Engineering in Medicine and Biology Society. 2008. p. 3475–8. 10.1109/IEMBS.2008.4649954 19163457

[64] Cesqui B, Aliboni S, Mazzoleni S, Carrozza MC, Posteraro F, Micera S. On the Use of Divergent Force Fields in Robot-Mediated Neurorehabilitation. In: 2008 2nd IEEE RAS EMBS International Conference on Biomedical Robotics and Biomechatronics. 2008. p. 854–61.

[65] Otaka E, Otaka Y, Kasuga S, Nishimoto A, Yamazaki K, Kawakami M (2015). Clinical usefulness and validity of robotic measures of reaching movement in hemiparetic stroke patients. J Neuroeng Rehabil.

[66] Wu C, Yang C, de Chen M, Lin K, Wu L (2013). Unilateral versus bilateral robot-assisted rehabilitation on arm-trunk control and functions post stroke: a randomized controlled trial. J Neuroeng Rehabil.

[67] Hazam Majid MS, Khairunizam W, Shahriman AB, Zunaidi I, Sahyudi BN, Zuradzman MR. EMG Feature Extractions for Upper-Limb Functional Movement During Rehabilitation. In: 2018 International Conference on Intelligent Informatics and Biomedical Sciences (ICIIBMS). 2018. p. 314–20.

[68] Dipietro L, Krebs HI, Fasoli SE, Volpe BT, Stein J, Bever C (2007). Changing motor synergies in chronic stroke. J Neurophysiol.

[69] Corti M, McGuirk TE, Wu SS, Patten C (2012). Differential effects of power training versus functional task practice on compensation and restoration of arm function after stroke. Neurorehabil Neural Repair.

[70] Grinyagin IV, Biryukova EV, Maier MA (2005). Kinematic and dynamic synergies of human precision-grip movements. J Neurophysiol.

[71] Osu R, Franklin DW, Kato H, Gomi H, Domen K, Yoshioka T (2002). Short- and long-term changes in joint co-contraction associated with motor learning as revealed from surface EMG. J Neurophysiol.

[72] Kisiel-Sajewicz K, Fang Y, Hrovat K, Yue GH, Siemionow V, Sun CK (2011). Weakening of synergist muscle coupling during reaching movement in stroke patients. Neurorehabil Neural Repair.

[73] Kim H, Miller LM, Fedulow I, Simkins M, Abrams GM, Byl N (2013). Kinematic data analysis for post-stroke patients following bilateral versus unilateral rehabilitation with an upper limb wearable robotic system. IEEE Trans Neural Syst Rehabil Eng.

[74] Kahn LE, Zygman ML, Rymer WZ, Reinkensmeyer DJ. Effect of robot-assisted and unassisted exercise on functional reaching in chronic hemiparesis. In: 2001 Conference Proceedings of the 23rd Annual International Conference of the IEEE Engineering in Medicine and Biology Society. 2001. p. 1344–7 vol.2.

[75] Knaut LA, Subramanian SK, McFadyen BJ, Bourbonnais D, Levin MF (2009). Kinematics of pointing movements made in a virtual versus a physical 3-dimensional environment in healthy and stroke subjects. Arch Phys Med Rehabil.

[76] Mace M, Guy S, Hussain A, Diane Playford E, Ward N, Balasubramanian S (2017). Validity of a sensor-based table-top platform to measure upper limb function. IEEE Int Conf Rehabil Robot.

[77] Hu XL, Tong K, Song R, Zheng XJ, Leung WWF (2009). A comparison between electromyography-driven robot and passive motion device on wrist rehabilitation for chronic stroke. Neurorehabil Neural Repair.

[78] Michaelsen SM, Jacobs S, Roby-Brami A, Levin MF (2004). Compensation for distal impairments of grasping in adults with hemiparesis. Exp Brain Res.

[79] Israely S, Leisman G, Carmeli E (2017). Improvement in arm and hand function after a stroke with task-oriented training. BMJ Case Rep.

[80] Zollo L, Gallotta E, Guglielmelli E, Sterzi S (2011). Robotic technologies and rehabilitation: new tools for upper-limb therapy and assessment in chronic stroke. Eur J Phys Rehabil Med.

[81] Mazzoleni S, Filippi M, Carrozza MC, Posteraro F, Puzzolante L, Falchi E (2011). Robot-aided therapy on the upper limb of subacute and chronic stroke patients: a biomechanical approach. IEEE Int Conf Rehabil Robot.

[82] Daly JJ, Hogan N, Perepezko EM, Krebs HI, Rogers JM, Goyal KS (2005). Response to upper-limb robotics and functional neuromuscular stimulation following stroke. J Rehabil Res Dev.

[83] Rohrer B, Fasoli S, Krebs HI, Hughes R, Volpe B, Frontera WR (2002). Movement smoothness changes during stroke recovery. J Neurosci.

[84] Longhi M, Merlo A, Prati P, Giacobbi M, Mazzoli D (2016). Instrumental indices for upper limb function assessment in stroke patients: a validation study. J Neuroeng Rehabil.

[85] Colombo R, Cusmano I, Sterpi I, Mazzone A, Delconte C, Pisano F (2014). Test-retest reliability of robotic assessment measures for the evaluation of upper limb recovery. IEEE Trans Neural Syst Rehabil Eng.

[86] Mazzoleni S, Posteraro F, Filippi M, Forte F, Micera S, Dario P (2011). Biomechanical assessment of reaching movements in post-stroke patients during a robot-aided rehabilitation. Appl Bionics Biomechanics.

[87] Chang JJ, Tung WL, Wu WL, Huang MH, Su FC (2007). Effects of robot-aided bilateral force-induced isokinetic arm training combined with conventional rehabilitation on arm motor function in patients with chronic stroke. Arch Phys Med Rehabil.

[88] Basteris A, Nijenhuis SM, Stienen AH, Buurke JH, Prange GB, Amirabdollahian F (2014). Training modalities in robot-mediated upper limb rehabilitation in stroke: a framework for classification based on a systematic review. J Neuroeng Rehabil.

[89] Dalla Gasperina S, Roveda L, Pedrocchi A, Braghin F, Gandolla M (2021). Review on patient-cooperative control strategies for upper-limb rehabilitation exoskeletons. Front Robot AI.

[90] Wearable Robots: Biomechatronic Exoskeletons | Wiley [Internet]. Wiley.com. [cited 2022 Apr 13]. Available from: https://www.wiley.com/en-us/Wearable+Robots%3A+Biomechatronic+Exoskeletons-p-9780470512944.

[91] Perry JC, Rosen J, Burns S (2007). Upper-limb powered exoskeleton design. IEEE/ASME Trans Mechatron.

[92] Wu G, van der Helm FCT, Veeger HEJD, Makhsous M, Van Roy P, Anglin C (2005). ISB recommendation on definitions of joint coordinate systems of various joints for the reporting of human joint motion–Part II: shoulder, elbow, wrist and hand. J Biomech.

[93] Georgarakis AM, Wolf P, Riener R. Simplifying Exosuits: Kinematic Couplings in the Upper Extremity during Daily Living Tasks. In: 2019 IEEE 16th International Conference on Rehabilitation Robotics (ICORR). 2019. p. 423–8. 10.1109/ICORR.2019.8779401 31374666

[94] Hermens HJ, Freriks B, Disselhorst-Klug C, Rau G (2000). Development of recommendations for SEMG sensors and sensor placement procedures. J Electromyogr Kinesiol.

[95] Organization WH, editor. International classification of functioning, disability and health: ICF. Geneva: World Health Organization; 2001.

[96] Masic I, Miokovic M, Muhamedagic B (2008). Evidence based medicine—new approaches and challenges. Acta Inform Med.

[97] Kanzler CM, Rinderknecht MD, Schwarz A, Lamers I, Gagnon C, Held JPO (2020). A data-driven framework for selecting and validating digital health metrics: use-case in neurological sensorimotor impairments. npj Digit Med..

[98] ICF Beginner’s Guide: Towards a Common Language for Functioning, Disability and Health [Internet]. [cited 2022 Jul 18]. Available from: https://www.who.int/publications/m/item/icf-beginner-s-guide-towards-a-common-language-for-functioning-disability-and-health.

[99] Definition of FEASIBILITY [Internet]. [cited 2022 Jun 20]. Available from: https://www.merriam-webster.com/dictionary/feasibility.

[100] Plesser HE (2018). Reproducibility vs. replicability: a brief history of a confused terminology. Front Neuroinform.

[101] Zbytniewska M, Kanzler CM, Jordan L, Salzmann C, Liepert J, Lambercy O (2021). Reliable and valid robot-assisted assessments of hand proprioceptive, motor and sensorimotor impairments after stroke. J NeuroEngineering Rehabil.

[102] Gandolla M, Antonietti A, Longatelli V, Pedrocchi A (2020). The effectiveness of wearable upper limb assistive devices in degenerative neuromuscular diseases: a systematic review and meta-analysis. Front Bioeng Biotechnol [Internet]..

